# Morphology and identification of the final instar nymphs of three cicadas (Hemiptera, Cicadidae) in Guanzhong Plain, China based on comparative morphometrics

**DOI:** 10.3897/zookeys.425.7897

**Published:** 2014-07-10

**Authors:** Zehai Hou, Qinglong Li, Cong Wei

**Affiliations:** 1 Key Laboratory of Plant Protection Resources and Pest Management, Ministry of Education, Entomological Museum, Northwest A&F University, Yangling, Shaanxi 712100, China

**Keywords:** Cicadoidea, immature stage, underground pest

## Abstract

The present investigation provides comparative morphometrics of the final instar nymphs of three dominant cicada species, i.e., *Cryptotympana atrata* (Fabricius), *Meimuna mongolica* (Distant) and *Platypleura kaempferi* (Fabricius), in Guanzhong Plain, China. Particularly, characters on the antennae, legs, and apex of abdomen of both males and females of these three species were investigated and analyzed. In addition, the numbers of hind tibial spines of the final instar nymphs of 21 representatives of Cicadoidea were compared. The results provide useful characteristics for nymph identification of related species and for further taxonomic and phylogenetic analysis of Cicadoidea.

## Introduction

Cicadas (Hemiptera, Cicadidae) are well known for their loud calling songs produced by male adults during summer (Young and Bennet-Clark 1995) and their long-term immature stage which is much longer than the adult stage, lasting several years underground (Boulard 1965, Pachas 1966, Logan 2006). During their subterranean lives from the first to final instars, cicada nymphs, burrowing through soil and feeding on xylem roots (White and Strehl 1978), may occasionally cause damage to their host plants. They have powerful forelegs modified for digging, and related morphological characters have been recognized for nymph identification in a few species by several authors (Boulard 1965, Pachas 1966, Hayashi 1976, Williams and Simon 1995, Ellingson et al. 2002). However, little information is available for nymph morphology or identification of most cicada species.

The cicadas *Cryptotympana atrata* (Fabricius), *Meimuna mongolica* (Distant) and *Platypleura kaempferi* (Fabricius), all belonging to the subfamily Cicadinae, are the three most dominant species in Guanzhong Plain of Shaanxi Province, China, which lies north of Qinling Mountains, the convergence zone of the Palaearctic and the Oriental regions and the natural boundary between northern and southern China. Like other cicadas, these three species, particularly *Cryptotympana atrata* (a dominant pest in apple orchards of northen China), can cause great harm including twig dieback in host plants when large numbers of females insert eggs into the stems of trees and, furthermore, injuries caused by the feeding of them usually go undetected since their nymphs are long-lived underground (Zhu et al. 2012). Previous studies on these cicadas were mainly focused on adult morphology and taxonomy (Chou et al. 1997), and the morphology or morphometrics of the final instar exuviae (Kato 1931, Hayashi 1974, 1975, 1987; Lee et al. 2012). Herein, we investigate the morphometrics of the final instar nymphs of these three cicadas, aiming to give a detailed description of the final instar nymphs, compare the gross morphology among different species, and provide more information for nymph identification and future investigation about their biology, ontogeny and ethology.

## Materials and methods

### Materials

All nymphs of the final instar were collected by digging beneath the woods, i.e., *Cryptotympana atrata* beneath *Populus tomentosa* Carr., *Platypleura kaempferi* beneath *Metasequoia glyptostroboides* Hu & Cheng, and *Meimuna mongolica* beneath *Pyrus xerophila* Yü on the campus of Northwest A&F University, Yangling, Shaanxi Province, China, from October to December, 2013. All captured nymphs were transferred alive to a beaker and anesthetized by chilling in a 4 °C refrigerator for morphological investigation. Exuviae and adult cicadas of the above three species were also collected on their host plants from June to July, 2013, respectively, aiming to confirm the identification of the final instar nymphs of each related species based on morphology. In addition, the nymphs of the final instar, exuviae and adult cicadas of *Subpsaltria yangi* Chen (belonging to the subfamily Tettigadinae) and *Karenia caelatata* Distant (belonging to the subfamily Cicadettinae) were also collected in the same way in Mts Helan, Ningxia Hui Autonomous Region, China, in June, 2012, and at Ningshan County in Mts Qinling, Shaanxi Province, China, in July and August, 2012, respectively. All the above mentioned materials and the exuviae of *Cicadetta shansiensis* (Esaki & Ishihara) deposited in the Entomological Museum of Northwest A&F University, China were examined, aiming to make a comparative morphological study on the hind tibial spines among these species and also other related species which have been investigated by some authors (Hayashi 1999; Maccagnan and Martinelli 2004, 2011; Logan and Connolly 2005).

### Methods

Nymphs were classified to sex by the developing genitalia at the apex of abdomen. For males, the several terminal abdominal segments of part materials were slightly extracted to show the 9^th^ abdominal sternite if necessary, which was partly concealed by the 10^th^ abdominal sternite. Observations of the morphological features were carried out using a Motic SMZ168 Stereoscopic Zoom Microscope. Photographs were taken with a scientific digital micrography system equipped with an Auto-montage imaging system and a Qimaging Retiga 2000R digital camera (CCD). Drawings were made with the aid of a camera lucida attached to the microscope.

**Figure 1.**
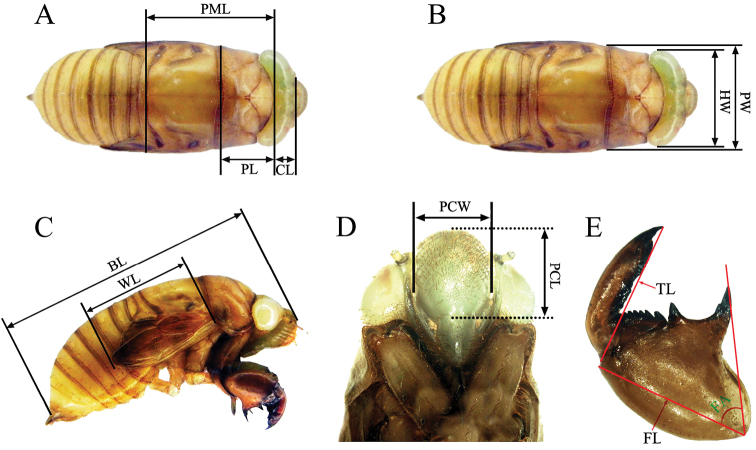
Morphological characters of final instar nymphs used for measurements. **BL** body length; **CL** crown length; **FA** femoral tooth angle; **FL** fore femur length; **HW** head width; **PCL** postclypeus length; **PCW** postclypeus width; **PML** pro-mesonotum length; **PL** pronotum length; **PW** pronotum width; **TL** fore tibiae length; **WL** wing length.

**Figure 2.**
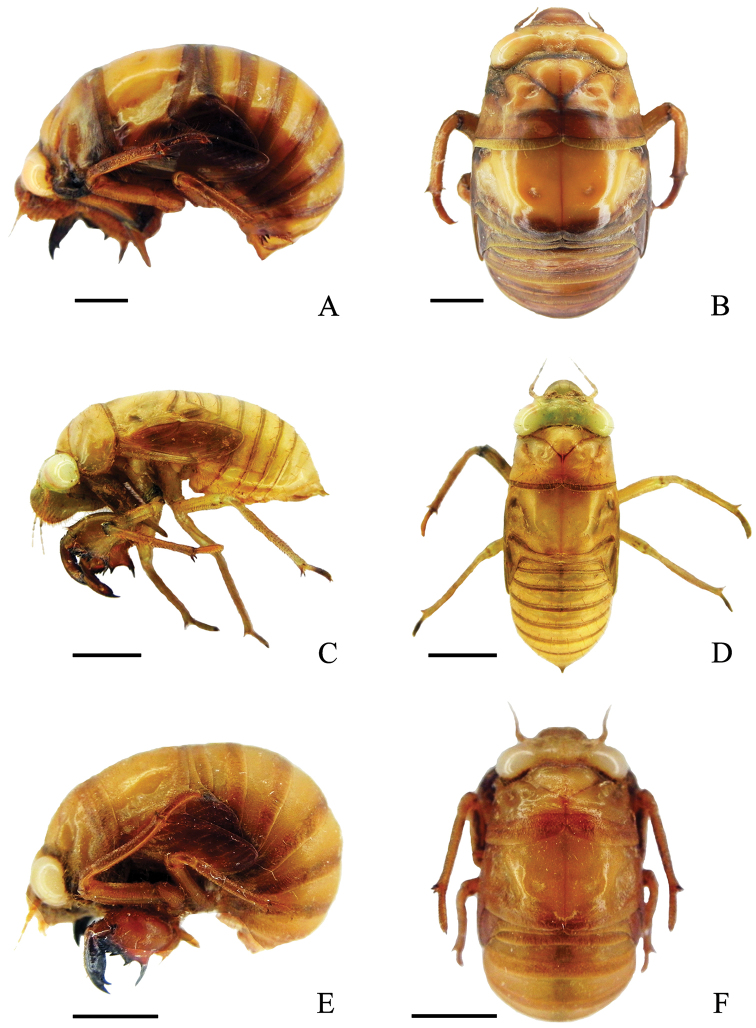
Final instar nymphs. **A** *Cryptotympana atrata*, lateral view of body **B** *Cryptotympana atrata*, dorsal view of body **C** *Meimuna mongolica*, lateral view of body **D** *Meimuna mongolica*, dorsal view of body **E** *Platypleura kaempferi*, lateral view of body **F** *Platypleura kaempferi*, dorsal view of body. Scale bars = 5.0 mm.

**Figure 3. F3:**
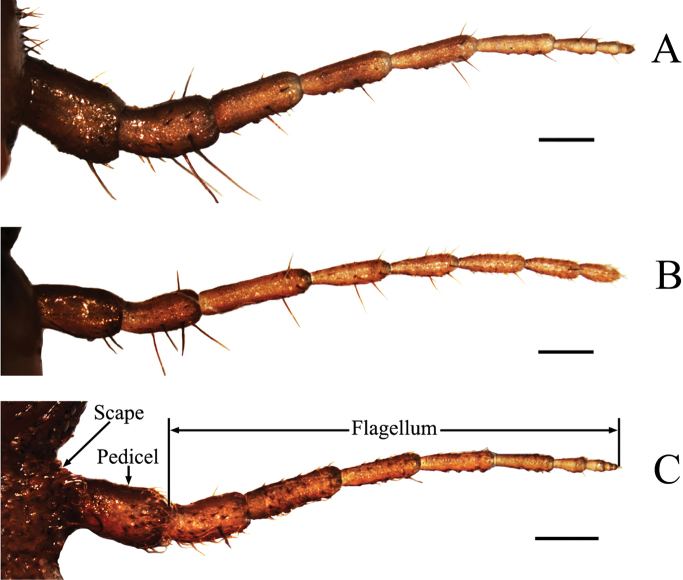
Antennae of final instar nymphs. **A** *Cryptotympana atrata* **B** *Meimuna mongolica* **C** *Platypleura kaempferi*. Scale bars = 0.5 mm.

**Figure 4. F4:**
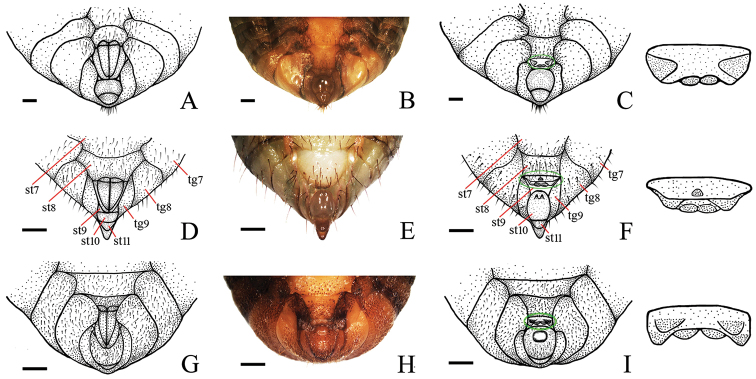
Abdominal apex in ventral view of final instar nymphs. **A** *Cryptotympana atrata*, female **B** and **C** *Cryptotympana atrata*, male **D** *Meimuna mongolica*, female **E** and **f** *Meimuna mongolica*, male **G** *Platypleura kaempferi*, female **H** and **I** *Platypleura Kaempferi*, male. Scale bars = 1.0 mm.

**Figure 5.**
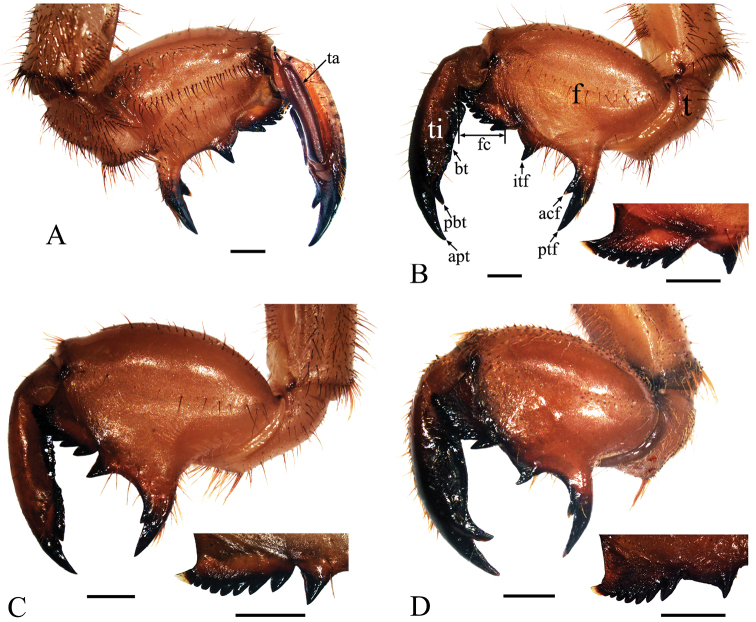
Left foreleg of final instar nymphs. **A** *Cryptotympana atrata*, inner view **B** *Cryptotympana atrata*, outer view **C** *Meimuna mongolica*, outer view **D** *Platypleura kaempferi*, outer view. acf, accessory tooth of femur; apt, apical tooth of tibia; bt, blade of tibia; f, femur; fc, femoral comb; itf, intermediate tooth of femur; pbt, point of blade of tibia; ptf, posterior tooth of femur; t, trochanter; ta, tarsus; ti, tibia. Scale bars = 1.0 mm

**Figure 6.**
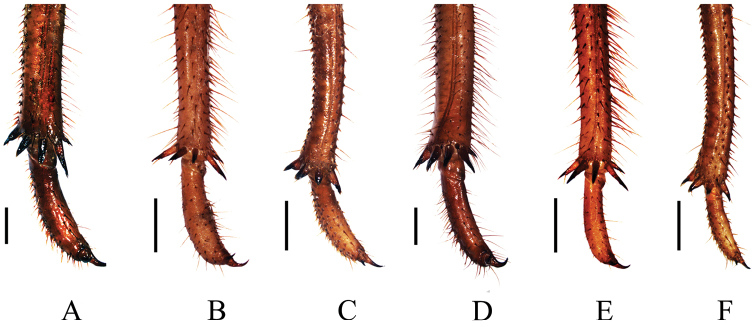
Spines at the apex of mid and hind tibiae of final instar nymphs. **A** mid tibia of *Cryptotympana atrata* **B** mid tibia of *Meimuna mongolica* **C** mid tibia of *Platypleura kaempferi* **D** hind tibia of *Cryptotympana atrata* **E** hind tibia of *Meimuna mongolica* **F** hind tibia of *Platypleura Kaempferi*. Scale bars = 1.0 mm.

Twenty individuals (10 males and 10 females, respectively) of each species were measured. The measurements are as follows: (1) crown length (CL) measured on dorsal view along its median line from frontoclypeal suture to posterior margin of head (Fig. 1A); (2) pronotum length (PL) measured on dorsal view along its median line (Fig. 1A); (3) pro-mesonotum length (PML) measured on dorsal view from pronotum to mesonotum along its median line (Fig. 1A); (4) head width (HW) measured on dorsal view from the outside of one compound eye to the other (Fig. 1B); (5) pronotum width (PW) measured on dorsal view at the posterior margin (Fig. 1B); (6) body length (BL) measured on lateral view from the apex of postclypeus to the distal margin of abdomen (Fig. 1C); (7) wing length (WL) measured on lateral view from rim of pronotum to apex of wing pocket (Fig. 1C); (8) postclypeus length (PCL) measured on front view from its suture with the anteclypeus to the frontoclypeal suture (Fig. 1D); (9) postclypeus width (PCW) measured on front view from one side of the outermost edge of frontoclypeal to the other (Fig. 1D); (10) fore femur length (FL) measured along the median line of its external side (Fig. 1E); (11) fore tibiae length (TL) measured along the median line of its external side (Fig. 1E); (12) femoral tooth angle (FA) measured between the longitudinal axis of femur and its posterior tooth (Fig. 1E).

Among these measurements, FA was measured using the Image Lab version 2.2.4.0 software (MCM Design, Hillerød, Denmark). The remaining measurements were taken using a vernier caliper with the accuracy of 0.02 mm.

Multivariate and univariate general linear model (GLM) analyses were conducted to determine whether morphological characters differed by species or sex. Statistical analyses were performed using SPSS 17.0.

The subfamily and tribal classification follows that of Moulds (2005). The terminology adopted to describe the structures of the foreleg was based on Duffels and Ewart (1988), and the femoral formula used to indicate the number and sequence of the teeth of fore femur was based on Maccagnan and Martinelli (2004).

## Results

### Morphology of the final instar nymph of *Cryptotympana atrata* (Fabricius, 1775)

#### Cryptotympana atrata

Taxon classificationAnimaliaHemipteraCicadidae

(Fabricius, 1775)

Figs 2 [Fig F3] [Fig F4] –5

Tettigonia atrata Fabricius, 1775: 681
Cicada atrata (Fabricius): Goeze 1778: 149
Tettigonia pustulata Fabricius, 1787: 266
Cicada nigra Olivier, 1790: 750
Fidicina bubo Walker, 1850: 82
Fidicina atrata (Fabricius): Walker 1850: 89
Cryptotympana atrata (Fabricius): Stål 1861: 613
Cryptotympana sinensis Distant, 1887: 415
Cryptotympana dubia Haupt, 1917: 229
Cryptotympana coreanus Kato, 1925: 13
Cryptotympana santoshonis Matsumura, 1927: 49
Cryptotympana wenchewensis Ouchi, 1938: 82
Cryptotypmana pustulata castanea Liu, 1940: 82
Cryptotympana pustulata fukienensis Liu, 1940: 82

##### Measurements

**(mm or degree).** Male (n = 10): BL 26.8 (23.5–31.5), PCL 5.2 (4.7–5.5), PCW 5.2 (4.9–5.7), CL 3.1 (2.9–3.3), HW 11.7 (10.9–12.1), PL 8.3 (7.7–8.7), PW 14.3 (13.6–15.2), PML 16.5 (15.5–17.3), WL 12.6 (11.9–13.1), FL 6.2 (5.9–6.5), TL 6.6 (6.1–7.1), FA 71.4 (69.5–73.2).

Female (n = 10): BL: 26.3 (23.3–29.7), PCL 5.2 (4.9–5.4), PCW 5.2 (5.0–5.3), CL 3.0 (2.8–3.2), HW 11.4 (10.5–12.1), PL 8.1 (7.3–8.7), PW 14.2 (13.6–15.1), PML 16.2 (15.2–17.0), WL 12.5 (11.5–13.1), FL 6.2 (5.7–6.5), TL 6.5 (6.0–7.0), FA 71.6 (69.4–73.4).

##### Description.

Body (Fig. 2A, B) dark brown, curved in lateral view, with sparse setae mainly on venter.

Head (Fig. 2B). Somewhat triangular in dorsal view; crown including white compound eyes about four times wider than long and about the same width as anterior margin of pronotum. Antenna brown, filiform. Postclypeus prominently swollen, covered with dense brown pile. Rostrum reaching to posterior coxae.

Thorax (Fig. 2A, B). Pronotum broad, paramedian and lateral fissures distinct, pronotal collar undeveloped, posterior margin distinctively concave medially in dorsal view. Mesonotum slightly wider than pronotum, with two small scutal depressions on disc. Metanotum very small. Fore wing bud developed, reaching to middle of 3^rd^ abdominal segment laterally; hind wing bud slightly developed.

Leg (Figs 5A, B, 6A, D). Generally dark brown. Fore femur with femoral formula 2-1-7: posterior tooth long and sharp, accessory tooth robust and sharp, intermediate tooth with projection in one of its sides; femoral comb usually with seven teeth, the first tooth about as large as the second tooth. Fore tibia arched, flattened laterally; apical tooth long; point of blade of tibia large and long, tooth-like, separated from apical tooth of blade by a strong incision. Apex of tibia with five spines in both mid and hind legs. Pretarsi of all legs well developed into a pair of claws of unequal sizes.

Abdomen (Fig. 4A–C). Size varying depends on the development of the nymph. In female, 8^th^ and 9^th^ sternites with two sharp posterior marginal protrusions. In male, 9^th^ sternite almost entirely concealed by 10^th^ sternite, four protrutions present on surface: a large triangular protrusion near lateral margins, respectively, and a pair of very small rounded protrusions on posterior margin; 10^th^ sternite smooth.

##### Variations of femoral comb.

Ten percent (2/20) of the individuals investigated with femoral comb with six teeth, instead of seven teeth.

### Morphology of the final instar nymph of *Meimuna mongolica* (Distant, 1881)

#### Meimuna mongolica

Taxon classificationAnimaliaHemipteraCicadidae

(Distant, 1881)

Figs 2 [Fig F3] [Fig F4] –5

Cosmopsaltria mongolica Distant, 1881: 638
Meimuna mongolica (Distant): Distant 1906: 66
Meimuna suigensis Matsumura, 1927: 1
Meimuna chosensis Matsumura, 1927: 52
Meimuna heijonis Matsumura, 1927: 52
Meimuna santoshonis Matsumura, 1927: 52
Meimuna gallosi Matsumura, 1927: 52

##### Measurements

**(mm or degree).** Male (n = 10): BL 19.8 (18.0–21.3), PCL 3.5 (3.2–3.7), PCW 3.2 (2.9–3.5), CL 2.4 (2.2–2.7), HW 7.2 (6.8–7.7), PL 4.6 (4.4–4.8), PW 7.8 (7.3–8.2), PML 9.4 (8.7–9.8), WL 8.7 (8.0–9.4), FL 4.8 (4.5–5.0), TL 4.6 (4.3–4.8), FA 61.4 (60.5–62.8).

Female (n = 10): BL 19.1 (17.9–20.9), PCL 3.3 (3.1–3.6), PCW 3.0 (2.7–3.2), CL 2.2 (2.0–2.6), HW 6.9 (6.8–7.2), PL 4.4 (4.2–4.7), PW 7.5 (7.2–8.3), PML 8.9 (8.4–9.7), WL 8.4 (8.0–8.9), FL 4.7 (4.5–4.9), TL 4.5 (4.2–4.7), FA 61.4 (60.1–63.2).

##### Description.

Body (Fig. 2C, D) pale brown, narrow and elongated, with setae scattered mainly on venter.

Head (Fig. 2D). Somewhat triangular in dorsal view; crown including white compound eyes about three times wider than long and slightly wider than anterior margin of pronotum. Antenna brown, filiform. Postclypeus prominently swollen, covered with dense brown pile. Rostrum reaching to posterior coxae.

Thorax (Fig. 2C, D). Pronotum broad, paramedian and lateral fissures distinct, pronotal collar undeveloped, posterior margin distinctively concave medially in dorsal view. Mesonotum slightly wider than pronotum, with two small scutal depressions on disc. Metanotum very small. Fore wing bud developed, reaching to middle of 4^th^ abdominal segment laterally, hind wing bud slightly developed.

Leg (Figs 5C, 6B, E). Generally dark brown. Fore femur with femoral formula 2-1-7 or 2-1-8: posterior tooth long and sharp, accessory tooth small, with apex somewhat blunt, intermediary tooth robust; femoral comb usually with seven or eight teeth, the first tooth about as large as the second tooth. Fore tibia arched, flattened laterally; apical tooth long; point of blade of tibia very small, toothlet-like, separated from apical tooth of blade by a very weak incision. Tibia with five apical spines in both mid and hind legs. Pretarsi of all legs well developed into a pair of claws of unequal sizes.

Abdomen (Fig. 4D–F). Size varying depending on the development of the nymph. In female, 8^th^ and 9^th^ sternites with two sharp posterior marginal protrusions. In male, 9^th^ sternite totally concealed by 10^th^ sternite, three protrusions present on surface: a smaller medial, coniform protrusion near anterior margin, and two larger rounded protrusions on posterior margin; 10^th^ sternite with two distinct projections adjacent to anterior margin.

##### Variations of femoral comb.

Forty percent (8/20) and 60% (12/20) of the individuals observed with seven and eight teeth on the femoral comb, respectively.

### Morphology of the final instar nymph of *Platypleura kaempferi* (Fabricius, 1794)

#### Platypleura kaempferi

Taxon classificationAnimaliaHemipteraCicadidae

(Fabricius, 1794)

Figs 2 [Fig F3] [Fig F4] –5

Tettigonia kaempferi Fabricius, 1794: 23
Cicada kaempferi (Fabricius): Walker 1850: 117
Platypleura kaempferi (Fabricius): Butler 1874: 189
Platypleura fuscangulis Butler, 1874: 189
Platypleura hyalino-limbata Signoret, 1881: 62
Platypleura repanda Uhler, 1896: 276 (nec Linnaeus)
Platypleura tsuchidai Kato, 1936: 758
Platypleura retracta Liu, 1940: 74

##### Measurements

**(mm or degree).** Male (n = 10): BL 19.0 (18.1–19.6), PCL 3.2 (3.1–3.5), PCW 3.3 (3.2–3.5), CL 2.2 (2.1–2.4), HW 7.5 (7.3–7.8), PL 4.7 (4.3–5.0), PW 9.2 (8.9–9.6), PML 10.0 (9.4–10.5), WL 7.4 (7.0–7.8), FL 4.1 (3.9–4.3), TL 4.5 (4.2–4.6), FA 77.1 (76.1–78.1).

Female (n = 10): BL 18.3 (17.7–18.6), PCL 3.1 (2.9–3.4), PCW 3.2 (3.1–3.4), CL 2.1 (2.0–2.2), HW 7.3 (7.1–7.7), PL 4.6 (4.2–4.9), PW 8.9 (8.6–9.2), PML 9.8 (8.9–10.2), WL 7.2 (6.9–7.6), FL 4.0 (3.8–4.2), TL 4.3 (4.1–4.5), FA 77.2 (76.4–78.8).

##### Description.

Body (Fig. 2E, F) brown, well curved in lateral view, with sparse setae mainly on venter.

Head (Fig. 2F). Somewhat triangular in dorsal view; crown including white compound eyes about three times wider than long and slightly wider than the anterior margin of the pronotum. Antenna brown, filiform. Postclypeus prominently swollen, covered with dense brown pile. Rostrum extending beyond posterior coxae.

Thorax (Fig. 2E, F). Pronotum broad, paramedian and lateral fissures distinct, pronotal collar developed, posterior margin distinctively concave medially in dorsal view. Mesonotum about as wide as pronotum, with two small scutal depressions on disc. Metanotum very small. Fore wing bud developed, reaching to middle of 3^rd^ abdominal segment laterally, hind wing bud slightly developed.

Leg (Figs 5D, 6C, F). Generally dark brown. Fore femur with femoral formula 2-1-7: posterior tooth long and sharp, accessory tooth robust and sharp, intermediate tooth with projection in one of its sides; femoral comb usually with seven teeth, the first tooth distinctly larger than the second tooth. Fore tibia arched, flattened laterally; apical tooth long; point of blade of tibia large and long, tooth-like, separated from apical tooth of blade by a strong incision. Apex of tibia usually with four spines in both mid and hind legs, but sometimes with a very small accessory spine. Pretarsi of all legs well developed into a pair of claws of unequal sizes.

Abdomen (Fig. 4G–I). Size varying depending on the development of the nymph. In female, 8^th^ and 9^th^ sternites with two sharp posterior marginal protrusions. In male, 9^th^ sternite almost entirely concealed by 10^th^ sternite, six protrusions present on surface: two triangular protrusions adjacent to posterior margin, and four rounded protrusions on posterior margin; 10^th^ sternite with a very large, medial, globular protrusion adjacent to anterior margin.

##### Variations of femoral comb.

Twenty percent (4/20) of the individuals studied with femoral comb with eight teeth, instead of seven teeth.

### Morphometrics and comparative morphology of antennae and forelegs among the three cicadas

The results showed that the species (Wilks’λ = 0, F = 817.078, hypothesis df = 24, error df = 90.000, P = 0) was a significant factor for all morphological characters, and that sex (Wilks’λ = 0.469, F = 4.241, hypothesis df =12, error df = 45.000, P = 0) was also significant for all characters, except for BL, FL and FA (Table 1).

**Table 1.** Results of univariate general linear model (GLM) for the morphological characters measured in cicada nymphs.

Source	Variable	df	M.S.	F	P
Species	BL	2	376.178	199.226	0.000
	PCL	2	25.226	887.718	0.000
	PCW	2	27.038	1002.519	0.000
	CL	2	4.289	219.129	0.000
	HW	2	121.916	1349.714	0.000
	PL	2	87.079	1291.314	0.000
	PW	2	245.548	1809.702	0.000
	PML	2	314.792	1488.336	0.000
	WL	2	150.091	963.221	0.000
	FL	2	24.449	782.357	0.000
	TL	2	27.888	631.693	0.000
	FA	2	1271.572	1386.341	0.000
Sex	BL	1	6.144	3.254	0.077
	PCL	1	0.131	4.598	0.036
	PCW	1	0.171	6.328	0.015
	CL	1	0.216	11.036	0.002
	HW	1	0.963	10.658	0.002
	PL	1	0.353	5.230	0.026
	PW	1	0.561	4.132	0.047
	PML	1	1.700	8.038	0.006
	WL	1	0.662	4.245	0.044
	FL	1	0.096	3.072	0.085
	TL	1	0.241	5.451	0.023
	FA	1	0.122	0.132	0.717
Error	BL	56	1.888		
	PCL	56	0.028		
	PCW	56	0.027		
	CL	56	0.020		
	HW	56	0.090		
	PL	56	0.067		
	PW	56	0.136		
	PML	56	0.212		
	WL	56	0.156		
	FL	56	0.031		
	TL	56	0.044		
	FA	56	0.917		

There are great similarities in the gross morphology of antennae among the final instar nymphs of these three cicadas, i.e., the scape inserts in an antennal fovea of the cranium at the side of the postclypeus near an anterior tentorial pit, which is partially concealed in the antennal fovea and dorsally covered by the overhanging ridge of the vertex. However, differences also exist and are mainly shown in two aspects of these species: i) the shape of antennae (tapering apically in *Cryptotympana atrata* and *Platypleura kaempferi* (Fig. 3A, C), but apical segment of flagellum in *Meimuna mongolica* with full length in similar diameter (Fig. 3B)); and ii) the number of flagellar segments (seven in *Meimuna mongolica* (Fig. 3B), eight in *Cryptotympana atrata* (Fig. 3A) and nine in *Platypleura kaempferi* (Fig. 3C)).

Similarly, though there are many similarities in the morphology of forelegs among the final instar nymphs of these three cicadas, differences also exist and are mainly shown in four aspects: i) the shape of the base of posterior tooth on femur (extraordinarily broadened in *Meimuna mongolica* (Fig. 5C), but moderately broadened in *Cryptotympana atrata* and *Platypleura kaempferi* (Fig. 5B, D)), ii) the shape of the base of intermediate tooth (extraordinarily broadened in *Cryptotympana atrata* and *Platypleura kaempferi* (Fig. 5B, D), but moderately broadened in *Meimuna mongolica* (Fig. 5C)); iii) the shape of blade of tibia (with a large and long tooth-like point of blade of tibia in *Cryptotympana atrata* and *Platypleura kaempferi* (Fig. 5B, D), but with a very small, reduced apical toothlet-like point of blade of tibia in *Meimuna mongolica* (Fig. 5C)); and iv) the femoral tooth angle (about 61°, 71° and 77° in *Meimuna mongolica*, *Cryptotympana atrata* and *Platypleura kaempferi*, respectively).

### Comparison of the number of hind tibial spines and condition of intermediate tooth in 21 representatives of Cicadoidea

In Tettigarctidae, three hind tibial spines were found in *Tettigarcta crinita* Distant. In Cicadidae, the numbers of hind tibial spines of the final instar nymphs of different species are usually the same within a subfamily, but vary among different subfamilies (Table 2). In Tettigadinae, three hind tibial spines were observed in *Subpsaltria yangi*. In Cicadettinae, except for *Cicadetta shansiensis* with three and *Karenia caelatata* with five hind tibial spines, four hind tibial spines were observed in all other species: *Amphipsalta cingulata* (Fabricius), *Amphipsalta zelandica* (Boisduval), *Kikihia ochrina* (Walker), *Kikihia scutellaris* (Walker), *Notopsalta sericea* (Walker), *Rhodopsalta cruentata* (Fabricius), and *Carineta fasciculata* (Germar). In Cicadinae, three hind tibial spines were found in the genus *Mogannia* Amyot & Audinet-Serville, four hind tibial spines in the genus *Nipponosemia* Kato, five hind tibial spines in the four investigated species (*Cryptotympana atrata* (Fig. 6D), *Meimuna mongolica* (Fig. 6E), *Quesada gigas* (Olivier) and *Fidicina mannifera* (Fabricius)), and four hind tibial spines with an additional small accessory spine internally were observed in the other four species, i.e., *Dorisiana drewseni* (Stål), *Dorisiana viridis* (Olivier), *Fidicinoides pronoe* (Walker) and *Platypleura kaempferi* (Fig. 6F).

**Table 2.** Number of hind tibial spines of the 21 representatives of Cicadoidea.

Species or genera	Tribes	Subfamilies	Families	Numbers	Sources
*Tettigarcta crinita* Distant, 1883	Tettigarctini	Tettigarctinae	Tettigarctidae	3	This study
*Subpsaltria yangi* Chen, 1943	Tibicinini	Tettigadinae	Cicadidae	3	This study
*Cicadetta shansiensis* (Esaki & Ishihara, 1950)	Cicadettini	Cicadettinae		3	This study
*Amphipsalta cingulata* (Fabricius, 1775)	Cicadettini			4	Logan and Connolly (2005)
*Amphipsalta zelandica* (Boisduval, 1835)	Cicadettini			4	Logan and Connolly (2005)
*Kikihia ochrina* (Walker, 1858)	Cicadettini			4	Logan and Connolly (2005)
*Kikihia scutellaris* (Walker, 1850)	Cicadettini			4	Logan and Connolly (2005)
*Notopsalta sericea* (Walker, 1850)	Cicadettini			4	Logan and Connolly (2005)
*Rhodopsalta cruentata* (Fabricius, 1775)	Cicadettini			4	Logan and Connolly (2005)
*Carineta fasciculata* (Germar, 1821)	Carinetini (= Sinosenini Boulard)			4	Maccagnan and Martinelli (2011)
*Karenia caelatata* Distant, 1888	Carinetini (= Sinosenini Boulard)			5	This study
*Mogannia* Amyot & Audinet-Serville, 1843	Cicadatrini (=Moganniini)	Cicadinae		3	Hayashi (1999)
*Nipponosemia* Kato, 1925	Cicadatrini (=Moganniini)			4	Hayashi (1999)
*Cryptotympana atrata* (Fabricius, 1775)	Cryptotympanini			5	This study
*Meimuna mongolica* (Distant, 1881)	Dundubiini			5	This study
*Quesada gigas* (Olivier, 1790)	Hyantiini			5	Maccagnan and Martinelli (2004)
*Fidicina mannifera* (Fabricius, 1803)	Fidicinini			5	Maccagnan and Martinelli (2011)
*Dorisiana drewseni* (Stål, 1854)	Fidicinini			4 with an additional small spine internally	Maccagnan and Martinelli (2011)
*Dorisiana viridis* (Olivier, 1790)	Fidicinini				Maccagnan and Martinelli (2011)
*Fidicinoides pronoe* (Walker, 1850)	Fidicinini				Maccagnan and Martinelli (2011)
*Platypleura kaempferi* (Fabricius, 1794)	Platypleurini				This study

There are some differences in the situation of an intermediate tooth on fore femur from the femoral comb, e.g., continuous from the femoral comb, or well separated from the comb. For example, the intermediate tooth is continuous from the femoral comb in *Meimuna mongolica* (Fig. 5C), *Cicadetta shansiensis* and *Karenia caelatata*; however, it is well separated from the comb in *Cryptotympana atrata* (Fig. 5B), *Platypleura kaempferi* (Fig. 5D) and *Subpsaltria yangi*.

## Discussion

The present study is the first to focus on the comparative morphology of the three cicadas in Guanzhong Plain. Cicadas usually have a long immature stage underground, which causes difficulties in their nymphal instar determination. A few species were reported to have five nymphal instars by some authors, e.g., *Mogannia minuta* Matsumura, *Magicicada septendecim* (Linnaeus), and *Diceroprocta apache* (Davis) (Hayashi 1976; Maier 1980; Ellingson et al. 2002); while a few other species were reported to have four instars by several authors, e.g., *Cryptotympana atrata* and *Leptopsalta yamashitai* (Esaki & Ishihara) (Hu et al. 1990; Kang et al. 2005). The number of nymphal instars in cicadas needs to be readdressed based on more investigations. Though major similarities shared by nymphs of different instars within a species, the nymphs of final instar can be easily distinguished from the remaining instar nymphs by the well developed and rounded eye-capsule, the developed wing buds, and the apex of abdomen. In addition, some characteristics of the final instar nymphs are preserved in their exuviae, e.g., the femoral tooth angle, the shapes of foreleg and postclypeus, etc., which are informative for the recognition of the final instar nymphs from the remaining instar nymphs within a species. However, nymphs and adults of cicadas within a species have significant morphological differences which are closely related to their different ecological niches (Li and Wei 2013), and cause difficulties in species identification of most cicada nymphs. In the present study we show that the final instar nymphs of cicadas can be distinguished from each other according to their morphology. Among which, the differences of antennae and the 9^th^ and 10^th^ sternites in males may be important characters for taxonomic and phylogenetic analysis. In addition, the number of the hind tibial spines of the final instar nymphs may be an important morphological characteristic in phylogenetic analysis, although the number of hind tibial spines may be variable within a group, e.g., number of hind tibial spines of the genera *Nipponosemia* and *Mogannia* in the tribe Cicadatrini (=Moganniini) are 4 and 3, respectively (Hayashi 1999). The number of hind tibial spines of the final instar nymphs of more taxa need to be investigated when more materials become available. Surprisingly, the number of the hind tibial spines of the final instar nymphs of *Karenia caelatata* is 5, which is different with that of other investigated members of Cicadettinae, but is consistent with that of some members belonging to the Cicadinae, e.g., *Cryptotympana atrata*, *Meimuna mongolica*, *Fidicina mannifera*, and *Quesada gigas*. Moulds (2005) and Boulard (2008) attributed the *Karenia* in the Cicadettinae (=Tibicininae *auct.*). However, the number of the hind tibial spines of the final instar nymphs together with some other characteristics of adults (e.g., metanotum distinctly concealed by the cruciform elevation on dorsal midline, uncus well developed with uncal lobes elongated apically, etc.) suggest that it seems more plausible to place this genus in the Cicadinae. The systematic placement of this genus needs further investigation.

Cicada nymphs could extend to 120 cm soil layer underground (Hugie and Passey 1963), though nymphs are most abundant within the well-rooted soil A horizon (eluvial horizon) and B horizon (illuvial horizon), typically between 10 and 30 cm from the ground surface (Luken and Kalisz 1989, O'Geen and Busacca 2001). However, burrowing depth of nymphal cicadas varies depending on the species. For example, our investigation showed that *Platypleura kaempferi* nymphs were located at 10–30 cm from the soil surface, being consistent with the results of Uematsu and Onogi (1980), and *Meimuna mongolica* nymphs were mainly distributed in 21–30 cm soil layer and could extend to 60 cm. Interestingly, significant differences were also observed in the forelegs of these two species. This suggests that the different burrowing depth in soil of the nymphs of different cicada species should be closely related to the development of their forelegs, and that the forelegs may provide promising characters for taxonomy and for future investigation about biology, ontogeny and ethology of related species.

## Supplementary Material

XML Treatment for Cryptotympana atrata

XML Treatment for Meimuna mongolica

XML Treatment for Platypleura kaempferi
